# Vision transformer architecture and applications in digital health: a tutorial and survey

**DOI:** 10.1186/s42492-023-00140-9

**Published:** 2023-07-10

**Authors:** Khalid Al-hammuri, Fayez Gebali, Awos Kanan, Ilamparithi Thirumarai Chelvan

**Affiliations:** 1 Electrical and Computer Engineering, University of Victoria, Victoria, V8W 2Y2 Canada; 2 Computer Engineering, Princess Sumaya University for Technology, Amman, 11941 Jordan

**Keywords:** Vision transformer, Digital health, Telehealth, Artificial intelligence, Medical imaging

## Abstract

The vision transformer (ViT) is a state-of-the-art architecture for image recognition tasks that plays an important role in digital health applications. Medical images account for 90% of the data in digital medicine applications. This article discusses the core foundations of the ViT architecture and its digital health applications. These applications include image segmentation, classification, detection, prediction, reconstruction, synthesis, and telehealth such as report generation and security. This article also presents a roadmap for implementing the ViT in digital health systems and discusses its limitations and challenges.

## Introduction

The coronavirus disease 2019 (COVID-19) pandemic demonstrated how artificial intelligence (AI) can help scale a system during emergencies with limited medical staff or existing safety concerns. AI algorithms are widely used in digital medicine solutions, mainly in image and text recognition tasks, to analyze medical data stored in clinical information systems and generate medical reports, and to assist in other technical operations such as robotic surgery. Among the various AI-assisted tools for analyzing medical images, the vision transformer (ViT) has emerged as a state-of-the-art algorithm that replaces or combines traditional techniques such as convolutional neural networks (CNNs). This article discusses the foundations and applications of the ViT in digital health.

The ViT [1, 2] is a type of neural network for image processing in computer vision tasks [3]. The backbone of the ViT is a self-attention mechanism typically used in natural language processing (NLP). The ViT was introduced to deal with the image processing limitations of common machine learning architectures such as CNNs [4], recurrent neural networks (RNNs) [5], and even the traditional transformers for language models [1, 6]. The ViT provides a strong representation of image features and trains data using fewer computational resources compared with CNNs [1].

CNNs are widely used in the machine learning field and are suitable for feature extraction in specific local regions. However, they are unable to capture the contextual relationship between image features in the global context. In contrast, the ViT applies an attention mechanism to understand the global relationships among features.

RNNs are used to obtain inferences about sequence-to-sequence relationships and memorizes some past data. However, they require a large memory and are unsuitable for extracting image features compared with the ViT or CNNs. Bidirectional encoder representations from transformers (BERT) was developed by Google to process language models [7] based on attention mechanisms [8]. BERT can efficiently process sequence-to-sequence models but requires a larger memory compared with an RNN or a long short-term memory (LSTM) [9].

BERT has limitations in processing imaging data and is effective only for flattened data in a sequential shape. To deal with this issue, the ViT splits images into patches then and flattens them for analysis as linear sequences [1] in a parallel processing mechanism.

The applications of the ViT in medical imaging include segmentation, classification, reconstruction, prognosis prediction, and telehealth (e.g., report generation and security).

The remainder of this paper is organized as follows. ViT architecture section describes the foundations of the ViT architecture. Applications of the ViT in digital health section presents an overview of the important applications of the ViT in medical imaging. Roadmap for implementing ViT section presents a roadmap for the end-to-end implementation of the ViT. Limitations and challenges of ViT in digital health section discusses the limitations and challenges of using the ViT, and Conclusions section concludes the paper.

## ViT architecture

**Fig. 1:**
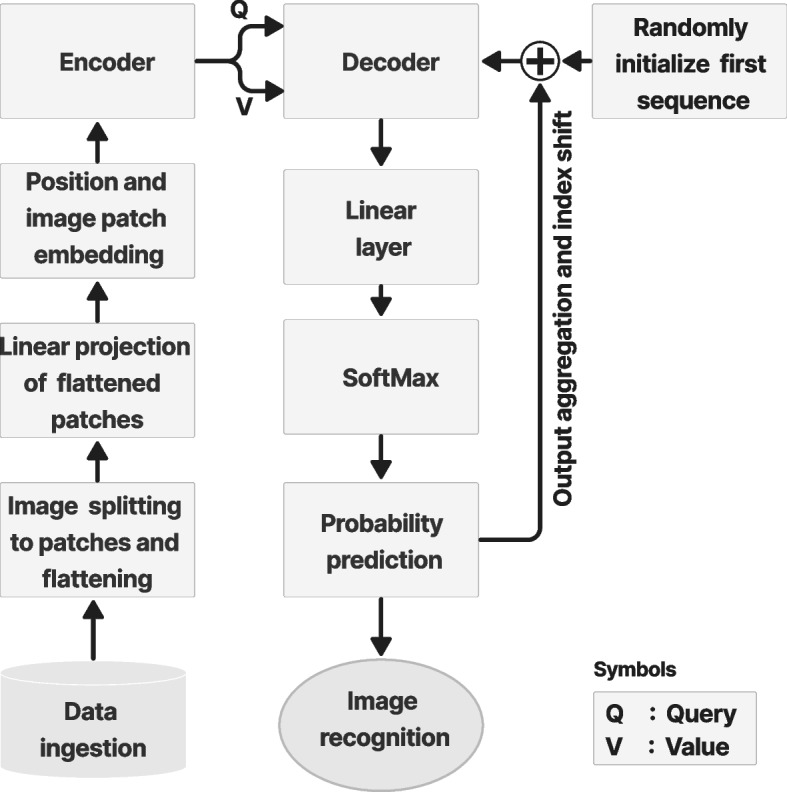
Transformer architecture [1]

This section discusses the core principles and foundations of the ViT based on the attention mechanism. The ViT architecture consists of a hierarchy of different functional blocks, which will be explained in the following subsections. Figure 1 shows the typical transformer architecture proposed by ref. [8] based on the attention mechanism.

Researchers have proposed various modifications for typical transformer designs [8] (Fig. 18 for a typical transformer architecture in Appendix) for applications other than NLP tasks. The changes focus on the design framework of encoder-decoder blocks in the transformer architecture. In vision tasks, the transformer splits the image into patches and flattens them into sequential forms to be processed like time-series data, which is more suited to the nature of transformers (Fig. 19 in Appendix). To ensure that an image can be reconstructed without any data loss, positional encoding was utilized for the embedded features in a vector shape. The embedded features were fed into the encoder for image classification and then classified by multilayer perception [1]. However, in the segmentation task, the transformer is combined with the CNN either in the encoder stage, similar to the TransUNet architecture (Fig. 20 in Appendix) [10], or in both the encoder and decoder stages, such as the Ds-TransUNet [11] (Fig. 21 in Appendix).

### Encoder architecture

**Fig. 2:**
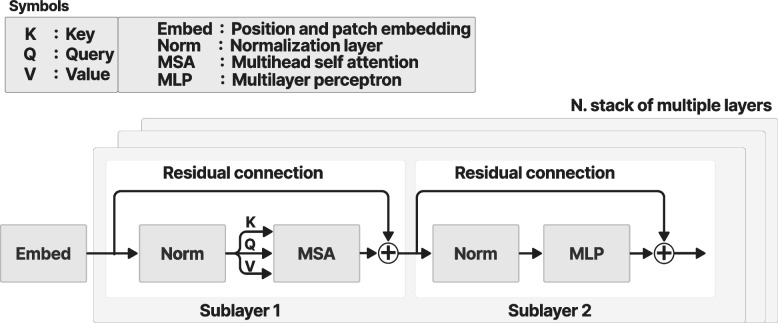
Encoder block in the transformer architecture [1]

Figure 2 shows a typical encoder architecture [8] that consists of a stack of *N* identical layers, with each layer containing two sublayers. The first sublayer performs the multihead self-attention (MSA), while the second sublayer normalizes the output of the first sublayer and feeds it into the multilayer perceptron (MLP), which is a type of feedforward network. See Appendix A.1 for an example of the transformer architecture in ref. [1].

### Image patches embedding

Owing to computer memory limitations, the simultaneous processing of an entire image is difficult. Therefore, the image is divided into different patches and processed sequentially. To conduct a detailed analysis of each image patch, each one was embedded into a set of feature values in the form of a vector.

The concept of image patch embedding in the ViT was inspired by the term ‘embedding’ in ref. [12]. The feature vectors were then graphically visualized in an embedding space. Visualizing the features in the embedding space is beneficial to identify the image patches with similar features [13]. The distance between each feature can be measured in the features map to determine the degree of similarity [14].

**Fig. 3:**
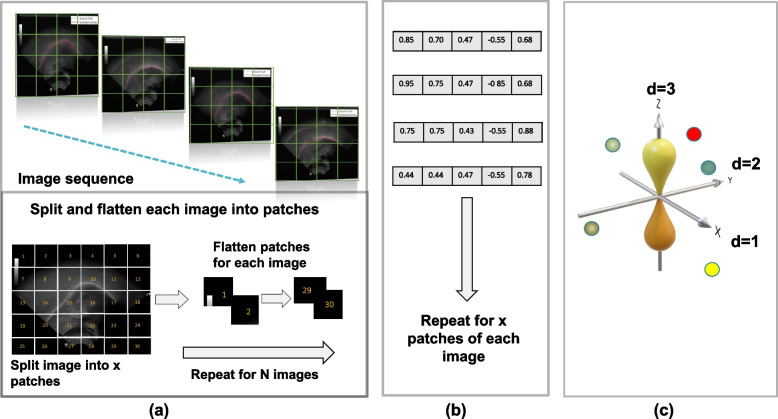
**a** Illustration of splitting ultrasound images into patches and flattening them in a linear sequence; **b** Image patch vectorization and linear projection; **c** Patch embedding in multidimensional space

Figure 3 shows the feature embedding process, which begins by creating an embedding layer from the embedding vectors of each input feature. Random embedding values are initially assigned and updated during training inside the embedding layer. During training, similar features become closer to each other in the embedding or latent space. This is important to classify or extract similar features. However, not knowing the position of each feature makes it difficult to determine the relationship between them. In medical imaging applications, positional encoding and feature embedding enable accurate feature selection in a specific-use case.

### Positional encoding

The transformer model has the advantage of simultaneously processing inputs in parallel, unlike the well-known LSTM algorithm [15, 16]. However, parallel processing is difficult because of the risk of information loss due to the inability to reconstruct the processed sequences in their original positions.

**Fig. 4:**
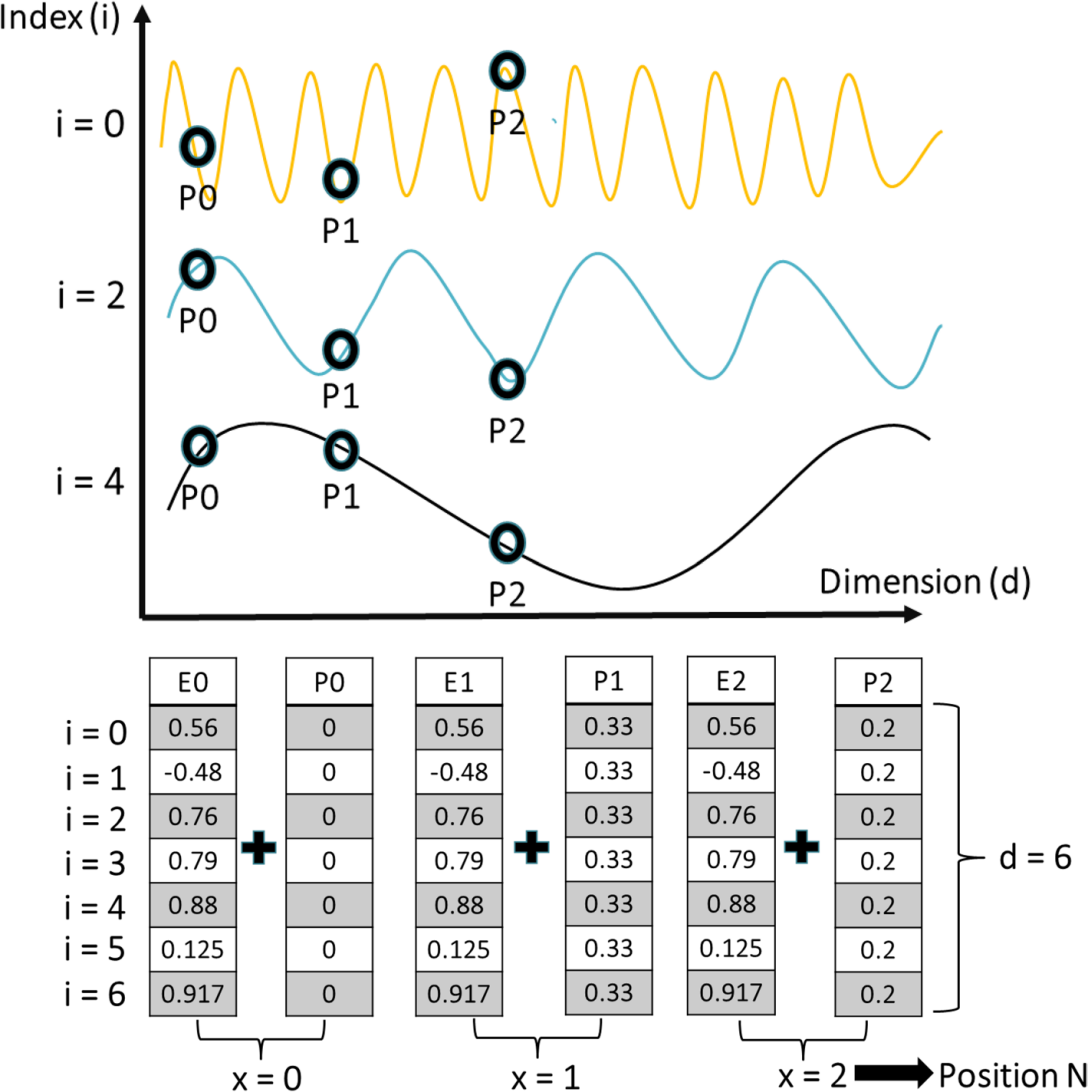
Positional encoding for the feature representations. Top: Sinusoidal representation for the positional encoding (P0-P3) at different indices and dimensions. Bottom: Vector representation for the positional encoding and feature embedding; P is the position encoding and E is the embedding vector

Figure 4 shows the positional encoding process for feature representation. Positional encoding was proposed to solve this problem and encode each feature vector to its accurate position [8, 17]. The feature vector and positional encoding values were added to form a new vector in the embedding space. In this study, sine and cosine functions were used as examples to derive the positional encoding values at different frequencies, expressed as Eqs.(1) and (2) [8], respectively.

1 $$P\left(x,2i\right)=\sin\left(\frac x{10000^\frac{2i}d}\right)$$

2 $$P\left(x,2i+1\right)=\cos\left(\frac x{10000^\frac{2i}d}\right)$$

where *P* is the positional encoding, *d* is the vector dimension, *x* is the position, and *i* is the index dimension. The sinusoidal function is beneficial for encoding the feature position in the embedding space using frequencies ranging from 2*π* to 10000. In Eqs. (1) and (2), the frequencies resembled the index dimension *i* [8].

### MSA

**Fig. 5:**
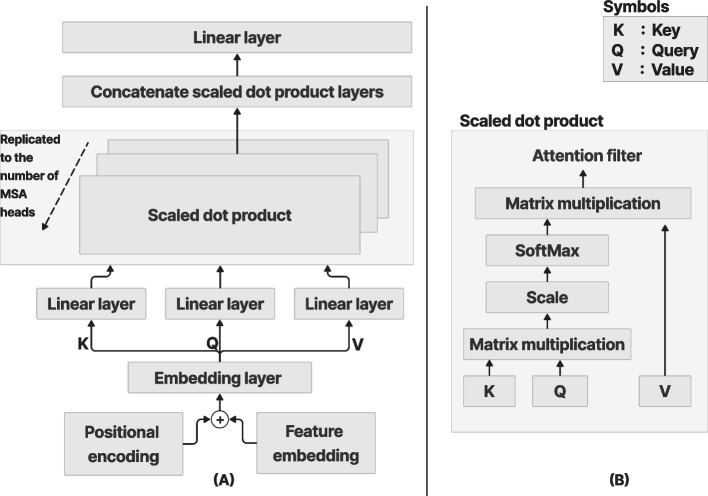
MSA process. **a** MSA process with several attention layers in parallel; **b** Scaled dot product [8]. The diagram flows upwards from the bottom according to the direction of the arrow

Figure 5 shows the MSA process, which calculates the weighted average of feature representations based on the similarity scores between pairs of representations. Given the input sequence *X* of *L* tokens or entries with the dimension *d*, *X* ∈ *R*^*L*×*d*^ was projected using three matrices: *W*_*K*_ ∈ *R*^*d*×*dk*^, *W*_*Q*_ ∈ *R*^*d*×*dq*^, and *W*_*V*_ ∈ *R*^*d*×*dv*^ with the same dimensions to derive the representation of the features. Equation (3) presents the formulas used to derive the Key (*K*), Query (*Q*), and Value (*V*).

3 $$K={XW}_{K}, Q={XW}_{Q},\,V={XW}_{V}$$

The final embedding layer that includes the position encoding was copied into the three linear layers *K*, *Q*, and *V*. To derive the similarity between the input features, matrix multiplication between *K* and *Q* was performed using self-attention. The output was then scaled and normalized using SoftMax. The self-attention [3] process is explained in the following steps:

1. Calculate the score from the input of *Q* and *K*.
  4 $$S=Q\,{K}^{T}$$
2. Normalize the score to stabilize the training.
  5 $${N}_{s}={S}^{\surd }\overline{d }$$
3. Calculate the probabilities of the normalized score using *SoftMax*.
  6 $$P=SoftMax(Ns)$$
4. Compute the self-attention filter by multiplying P and V.
  7 $$Self-attention=PV$$

The multiplication of the outputs of *K* and *Q* were scaled by the square root of the input vector dimension, and then normalized by the SoftMax function to generate the probabilities. Equation (8) presents the SoftMax function, where *x* is the input data point. Equation (9) computes the attention filter.

8 $$SoftMax({x}_{i})= \frac{\mathrm{exp}({x}_{i})}{ \sum_j \mathrm{exp}({x}_{j})}$$

9 $$Self-attention\left(Q, K, V\right)=SoftMax\left(\frac{Q \cdot {K}^{T}}{\overline{\surd d} }\right). V$$

The output probabilities from SoftMax and the value layer were multiplied to obtain the desired output with emphasis on the desired features to filter out unnecessary data. The principle behind a multihead is to concatenate the results of different attention filters, with each one focusing on the desired features. The self-attention process is repeated multiple times to form the MSA. The final output of the concatenated MSA was passed through a linear layer and resized to a single head. Equation (10) presents the *MSA* formula.

10 $$MSA\left(Q,K,V\right)=C(h_1,\;\dots\;,h_n)W_0$$

where *C* is the concatenation of the multiheads; *W*_0_ is the projection weight; *Q*, *K* and *V* denote the Query, Key and Value, respectively; and *h* resembles each head in the self-attention process and was replicated *n* times. The number of replications was dependent on the amount of attention or the desired features needed to extract the required information. Figure 5 shows the MSA process in the ViT architecture. Detailed information on the scaled dot products between *K*, *Q*, and *V* are also presented.

### Layer normalization and residual connections

A residual connection is required to directly feed the output from the position encoding layer into the normalization layer by bypassing the MSA layer [18]. The residual connection is essential for knowledge preservation and to avoid vanishing gradient problems [19, 20]. The MSA layer is vital for extracting useful features from the input. However, it could also lead to the disregard of helpful information of lesser weight in the attention filter. Minimizing the value of the feature weight may cause a vanishing gradient during the model training stage. A vanishing gradient occurs when the gradient of the loss function is depleted and becomes almost or equal to zero while optimizing the weight in the backpropagation algorithm. The residual connection directly feeds information from the initial layers into the layers at the end of the neural network to preserve features and retain important information.

The add and normalize layer [21] combine the input from the position encoding and MSA layers, and then normalize them. The normalization layer is essential during training to speed up and stabilize the loss convergence. Normalization can be achieved by standardizing the activation of neurons along the axis of the features. Equations (11) and (12) are the statistical components of layer normalization over all the hidden units in the same layer [21].

11 $${\mu }^{l}=\frac{1}{H}(\sum_{i=1}\nolimits^{H}{a}_{i}^{l})$$

12 $${\sigma }^{l}=\sqrt{\frac{1}{H}\sum_{i=1}\nolimits^{H}{\left({a}_{i}^{l}-{\mu }^{l}\right)}^{2}}$$

where ${a}_{i}^{l}$ is the normalized value of the sum of the input features along the *i*^*th*^ hidden units in the *l*^*th*^ layers. *H* is the total number of hidden units in the layer. *µ* is the mean or average values of features along the axis in the normalization layer, *σ* is the standard deviation of the values of the features along the axis.

### MLP

**Fig. 6:**
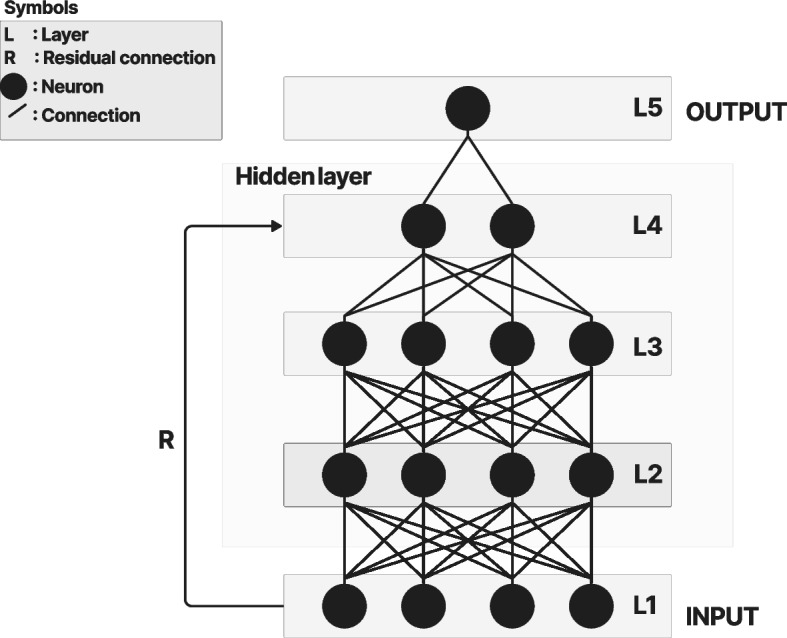
MLP

Figure 6 shows the MLP diagram, which is part of the ViT architecture. The MLP is a feedforward artificial neural network that combines a series of fully connected layers including the input, one or more hidden layers in the middle, and the output [22].

Fully connected layers are a type of layer in which the output of each neuron is connected to all the neurons in the next hidden layer. The diagram shows that each neuron from the layer in the feedforward neural network is connected to all the neurons in the next layer through an activation function. The residual connection preserves the knowledge from the initial layers and minimizes the vanishing gradient problem. Typical MLP layers include the input, output, and hidden layers.

### Decoder and mask MSA

**Fig. 7:**
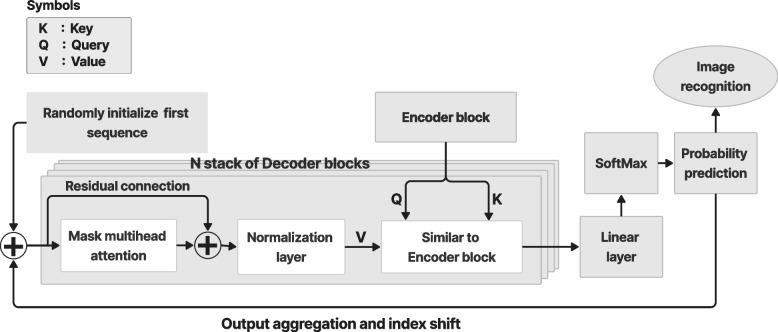
Decoder and mask multihead attention block to produce the final image

Figure 7 shows the decoder and mask MSA in the ViT architecture used to extract the final image. The decoder was stacked for *N* layers, the same as the number of encoder layers. The decoder includes the same sublayers as the encoder and mask MSA stacked on them. The mask MSA works similarly as the MSA, but focuses on the desired features in position *i* and ignores the undesirable features from the embedding layer by using the mask-only features before *i*. This is important to obtain an inference from the relationship between different features in the embedding space and a prediction from the features relevant to the desired position.

The decoder obtains the *V*, *Q*, and *K* as inputs. The *V* was obtained from the previous embedding space, while *Q* and *K* were obtained from the encoder output. There are other MSA and normalization layers inside the decoder, which is common in ViT designs. Despite modifications to the decoder-encoder design, the core principle remains the same. The different architectures for different applications are explained in Applications of the ViT in digital health section.

In the image recognition task, the decoder output was flattened as a linear or dense layer. Then, SoftMax was used to derive the probability of the weight of each neuron in the dense layer. The final probability was used to classify or segment the features based on the training data to detect the final object or image.

## Applications of the ViT in digital health

Computer vision and machine learning algorithms have been employed in recent medical studies on brain and breast tumors [23, 24], histopathology [25], speech recognition [26, 27], rheumatology [28], automatic captioning [29], endoscopy [30], fundus imaging [31], and telemedicine [32]. The ViT has emerged as the state-of-the-art in AI-based algorithms that use computer vision and machine learning for digital health solutions.

**Fig. 8:**
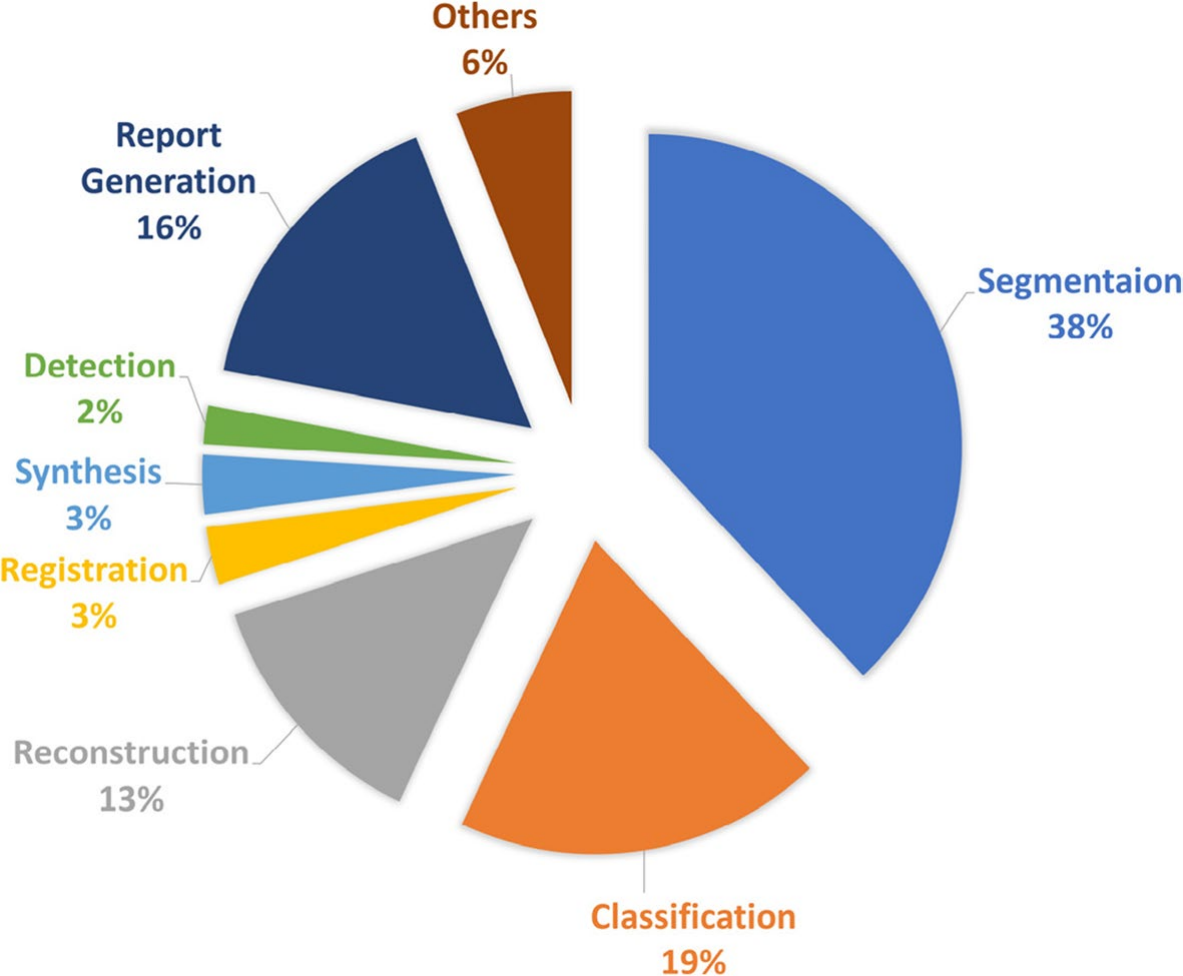
Distribution of medical imaging applications of the ViT according to the survey [33]

Figure 8 shows the distribution of ViT applications in the medical field. These include medical segmentation, detection, classification, report generation, registration, prognosis prediction, and telehealth.

### Applications of ViT in medical image segmentation

TransUNet [10] is one of the earliest attempts to apply the ViT in medical imaging segmentation by combining it with the UNet [34] architecture. UNet is well known in the area of biomedical image segmentation. It is efficient in object segmentation tasks and can preserve the quality of fine image details after reconstruction. The UNet inherited the localization ability of a CNN for feature extraction. Although localization is essential in a segmentation task, it has limitations in processing sequence-to-sequence image frames or extracting global features within the same image outside a specific region. In contrast, the ViT has the advantages of processing sequence-to-sequence features and extracting the global relationships between them. However, the ViT has limitations in feature localization compared with CNNs. TransUNet proposed a robust architecture that combined the capabilities of the ViT and UNet in a single model.

**Fig. 9:**
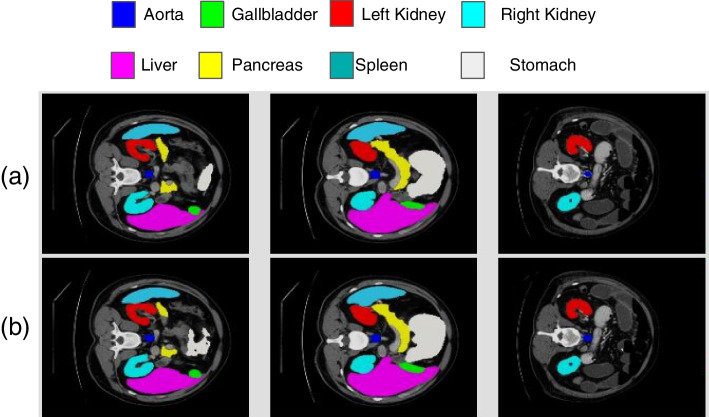
Comparison of TransUNet and GT using output segmentation results of different organs: **a** GT (expert reference) and **b** TransUNet [10]

TransUNet is a powerful tool for multiorgan segmentation. Segmenting different objects is essential to analyze complex structures in magnetic resonance imaging (MRI) and computed tomography (CT) images. Figure 9 shows an example of image segmentation of the abdomen in a CT scan using TransUNet, which was compared with ground truth (GT) images to validate the results.

To further improve the TransUNet architecture, a Dual-TransUNet was implemented in ref. [11]. The main difference is that the Dual-TransUNet used the transformer in the encoder to extract features and the decoder to reconstruct the desired image, while the TransUNet only used the transformer in the encoder stage. The Swin transformer [35] is another architecture for implementing the ViT in combination with Unet [34, 36] in medical imaging.

The ViT was also used in iSegFormer [37], which was proposed for the interactive segmentation of three-dimensional (3D) MRI images of the knee. The 3D UX-net [38] could segment brain tissues from the entire body in an MRI scan. UNesT [39] developed a hierarchical transformer using local spatial representation for brain, kidney, and abdominal multiorgan image segmentation. Similarly, the NestedFormer [40] was proposed to segment brain tumors in MRI images.

RECIST [41] used the ViT to automatically segment brain tumors to measure the size of the lesions in CT images. GT U-Net [42] was used for tooth therapy by segmenting the root canal in X-ray images. Colorectal cancer (CRC) images were segmented by the fully convolutional network (FCN) transformer [43] during a colonoscopy. The ViT was also used in the TraSeTR [44] to assist in robotic surgery by segmenting the image and generating instructions based on previous knowledge. Table 1 lists examples of ViT applications in medical image segmentation.

**Table 1:** Examples of ViT applications in medical image segmentation

Method	Category	Medical application
TransUnet [10]	MRI, CT	CT and MRI cardiac segmentation
Dual-TransUnet [11]	Microscopy	Skin lesion analysis [45]; gland segmentation in histology [46]; nuclei in divergent images [47]
Swin-Unet [35]	CT	Abdominal multiorgan segmentation
iSegFormer [37]	3D MRI	Knee image segmentation
3D UX-net [38]	3D MRI	Brain tissue segmentation
UNesT [39]	MRI, CT	Abdominal multiorgan segmentation + kidney segmentation + whole brain segmentation
NestedFormer [40]	MRI	Brain tumor segmentation
RECIST [41]	CT	Automatic tumor segmentation and diameter size prediction
GT U-Net [42]	X-ray	Tooth therapy: root canal segmentation
FCN-transformer [43]	Colonoscopy	CRC segmentation
TraSeTR [44]	Endoscopy	Robot-assisted surgery

### Applications of ViT in medical image detection

Image detection plays a key role in digital health and imaging analysis to identify objects in complex structures and share that information within the healthcare information system for further analysis. This is important to measure the cell size and count the number of suspicious objects or malignant tissues.

Object detection is essential in cancer screening when cell labeling or classification is difficult, and a careful analysis is required to identify cancers. The detection transformer (DETR) was proposed to detect lymphoproliferative diseases in MRI T2 images [48]. In MRI scans, the metastatic lymph nodes are small and difficult to identify. The application of the DETR can reduce false positives as well as improve the precision and sensitivity by 65.41% and 91.66%, respectively.

The convolutional transformer (COTR) [49] detects polyp lesions in colonoscopy images to diagnose CRC, which has the second highest cancer-related mortality risk worldwide. The COTR architecture employs a CNN for feature extraction and convergence acceleration. A transformer encoder is used to encode and recalibrate the features, a transformer decoder for object querying, and a feedforward network for object detection.

Global lesion detection in CT scans was performed using a slice attention transformer (SATr) [50]. The backbone of the SATr is a combination of convolution and transformer attention that detects log-distance feature dependencies while preserving the local features.

Lung nodule detection was investigated using an unsupervised contrastive learning-based transformer (UCLT) [51]. Lung nodules are small cancerous masses that are difficult to detect in complex lung structures because of their size. This study harnessed contrastive learning (CL) and the ViT to break down the volume of CT images into small patches of non-overlapping cubes, and extract the embedded features for processing using the transformer attention mechanism.

To predict the hemorrhage category of brain injuries in CT scans, a transformer-based architecture was used for intracranial hemorrhage detection (IHD) [52]. Table 2 lists examples of ViT applications in image classification.

**Table 2:** Examples of ViT applications in medical image detection

Method	Category	Medical application
DETR [48]	MRI	Lymphoproliferative diseases detection
COTR [49]	Colonoscopy	CRC detection
SATr [50]	CT	Universal lesion detection
UCLT [51]	CT	Lung nodule detection
IHD [52]	CT	Brain injury hemorrhage detection

### Applications of ViT in medical image classification

Classification is an important digital health solution in medical imaging analysis that helps medical practitioners identify objects within a complex structure to immediately categorize medical cases. Utilizing AI while working in remote areas and using telehealth systems with limited medical resources ensures the accuracy of final clinical decisions. The importance of AI emerged during the pandemic when the pressure on healthcare systems exceeded the capacity of the healthcare infrastructure. The ViT has different applications in medical imaging classification.

**Fig. 10:**
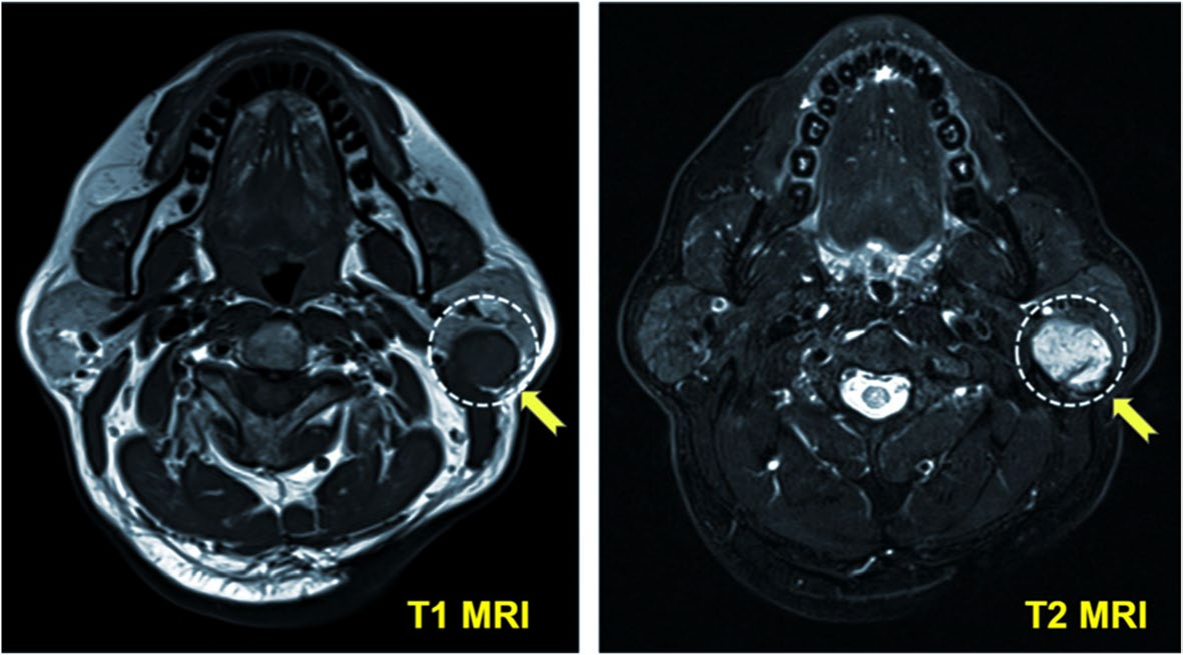
Example of using the ViT for tumor classification in MRI images using TransMed [53]. The tumor is enclosed by the dashed circle indicated by the yellow arrow

TransMed [53] uses a combination of the ViT and a CNN to classify multimodal data for medical analysis. The classification system includes disease and lesion identification. Figure 10 shows an example of the application of TransMed in image classification.

Shoulder implant manufacturers [54] use a transformer in orthopedic applications to assist in shoulder replacement surgery with artificial implants and joints. Before surgery, shoulder X-ray images were used to detect and classify the shoulder implant manufacturer vendor to determine the required accessories. The GasHis-transformer [55] is a multiscale visual transformer for detecting and classifying gastric cancer images using histopathological images of hematoxylin and eosin obtained by a microscope. Table 3 lists examples of ViT applications in image classification.

**Table 3:** Examples of ViT applications in medical image classification

Method	Category	Medical application
TransMed [53]	MRI	Multi-modal classification: disease classification, lesion identification
Shoulder implant manufacture [54]	X-ray	Orthopedics: Shoulder implant manufacture classification
GasHis-transformer [55]	Histopathology microscopic images	Gastric cancer classification and detection
Multi-scale cytopathology [56]	Cytopathological images	Cervical cancer classification
Brain metastases classification [57]	MRI	Classification of the brain tumor of central nervous system
ScoreNet [58]	Histology Datasets of haematoxylin + eosin	Breast cancer classification
RadioTransformer [59]	X-ray	COVID-19 classification using chest X-ray images
TractoFormer [60]	Diffusion MRI	Nerve tracts modelling and 3D fiber representation

A comparative analysis of cervical cancer classifications using various deep learning (DL) algorithms, including the ViT, was conducted using cytopathological images [56]. A transformer-based model was used in brain metastases classification [57] from an MRI of the brain. Brain metastases are among the main causes of malignant tumors in the central nervous system [61]. ScoreNet [58] is a transformer-based model that classifies breast cancer using histopathology images. RadioTransformer [59] classifies COVID-19 cases based on chest X-rays. TractoFormer [60] classifies brain images based on tractography, which is a 3D model of the brain nerve tracts using diffusion MRI. TractoFormer discriminates between 3D fiber spatial relationships. It has proven to be accurate in classifying patients with schizophrenia vs controls.

### Applications of ViT in medical imaging prognosis predication

**Fig. 11:**
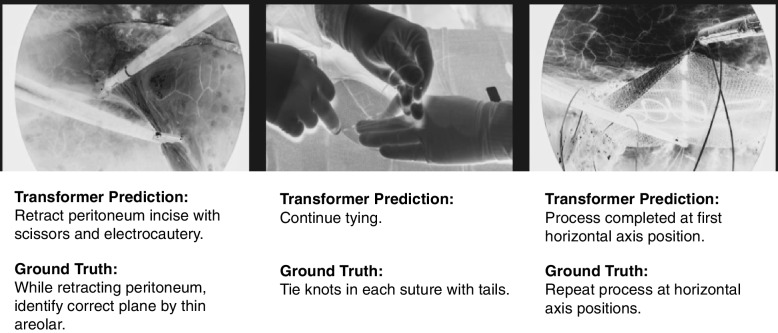
Examples of using ViT for surgical instruction prediction. Transformer prediction is based on the SIGT method [62]. GT is used as a reference for comparison and validation

The ability of the ViT to analyze time-series sequence data and obtain insights from previous data allows the prediction of future behaviors or patterns. In medical imaging, it is important to help healthcare practitioners predict the effects of diseases or cancers to treat them before they spread. Figure 11 shows the use of the transformer for surgical instructions, which are also implemented in Surgical Instruction Generation Transformer (SIGT) algorithm for surgical robots [62]. The algorithm used the ViT to analyze the visual scene during surgery and update the reinforcement learning (RL), reward, and status to predict the instructions for the robot.

The Sig-Former [63] can predict surgical instructions during an operation using the transformer attention mechanism to analyze the input image. The dataset includes images acquired during surgeries such as laparoscopic sleeve gastrectomy and laparoscopic ventral hernia repair.

The 3D Shuffle Mixer [64] analyzes 3D volumetric images from CT and MRI using context-aware dense predictions for different diseases, such as hemorrhagic stroke, abdominal CT images, and brain tumors.

Graph-based transformer models [65] predict genetic alteration. Ultrasound recordings are used for fetal weight prediction by the residual transformer model [66]. CLIMAT [67] forecasts the trajectory of knee osteoarthritis based on X-ray images from specialized radiologists. Table 4 lists examples of ViT applications in medical image prediction.

**Table 4:** Examples of ViT applications in medical image prediction

Method	Category	Medical application
3D-SMx [64]	3D (MRI, CT)	Context-aware dense prediction for different diseases that includes hemorrhagic stroke, abdominal CT images, brain tumor
GBT [65]	Cancer genome (TCGA)	Computation pathology: genetic alteration
RTM [66]	Ultrasound	Fetal weigh at birth prediction
CLIMAT [67]	X-ray	Forecasts knee osteoarthritis trajectory
Sig-Former [63]	Laparoscopy	Surgical instructions prediction
SIGT [62]	Robot camera	Surgical instruction prediction and image captioning

### Applications of ViT in image reconstruction and synthesis

After acquiring data from medical imaging modalities such as MRI, CT, and digital X-ray, the images are stored as raw data in an unstructured format. To make this raw data readable, a reconstruction process is applied to retrieve images without any loss. However, this process is computationally expensive because of the size and complexity of reconstruction algorithms. The use of DL significantly improves the reconstruction performance by enhancing the preservation of fine image details within a reconstruction time of a few seconds. In contrast, traditional techniques such as image reconstruction using compressed sensing require more time [68]. Reconstructing magnetic resonance images is a challenge because of the size, complexity, and sparsity of the K-space matrix, in which the raw images are stored in the frequency domain.

**Fig. 12:**
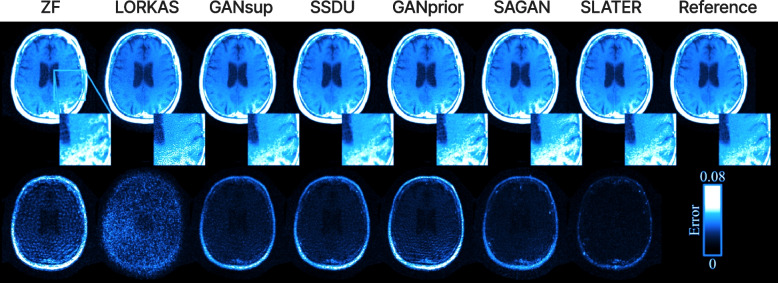
Top: Different reconstruction methods from *T*_1_ weighted acquisition of the fast MRI using different methods. ZF is a traditional Fourier method [70]. LORKAS [71, 72], GAN_*sub*_ [73], SSDU [74], GAN_*prior*_ [75], and SAGAN [76] are generative adversarial network (GAN) reconstruction-based methods. SLATER is a ViT-based method [69]. Bottom: Reconstruction error map [69]

SLATER [69] is a zero-shot adversarial transformer that performs the unsupervised reconstruction MRI images. SLATER maps the noise and latent representation to the MR coil-combined images. To maximize the consistency of the images, the operator input and maximum optimized prior information were combined using a zero-shot reconstruction algorithm. Figure 12 shows different methods for reconstructing fast MRI and the reconstruction error map using SLATER (ViT-based method) from *T*_1_ weighted images. These were then compared with other techniques based on non-ViT methods.

The Task Transformer (*T*^2^*Net*) [77] proposed an architecture to simultaneously reconstruct and enhance images using a super-resolution method for MRI. The *T* ^2^*Net* process can be divided into two parts. First, two CNN subtasks were used to extract domain-specific features. Second, *T* ^2^*Net* was embedded and the relationship between the two subtasks was synthesized. ReconFormer addresses the problem of under sampled K-space data by utilizing recurrent pyramid transformer layers to rapidly and efficiently retrieve the data [78]. Transformer-based methods for fast MRI reconstruction were evaluated in ref. [79]. The results showed that the combination of GANs and ViT achieved the best performance, i.e., a 30% improvement over standard methods such as the Swin transformer. Table 5 lists examples of ViT applications in image reconstruction.

**Table 5:** Examples of ViT applications in medical image reconstruction

Method	Category	Medical application
SLATER [69]	MRI	MRI unsupervised reconstruction
*T* ^2^*Net* [77]	MRI	Image reconstruction and super-resolution enhancement
ReconFormer[78], FastMRIRecon [79]	MRI	Accelerated MRI reconstruction
E-DSSR [80]	Endoscopy	Surgical robot scene reconstruction
DuTrans [81], MIST-net [82]	CT	CT sinograms reconstruction

A ViT-based (stereo transformer) was utilized in efficient dynamic surgical scene reconstruction [80] to reconstruct a robotic surgery scene acquired by an endoscope. This application is essential for surgical education, robotic guidance, and context-aware representation.

DuTrans adopted a Swin transformer as the core of their architectural design to reconstruct the sinograms of CT scans from the attenuation coefficient of the Hounsfield unit [81, 83]. The accurate reconstruction of CT scans is essential to obtain high-quality images, reduce radiation doses, and distinguish fine details to facilitate the early detection of cancers.

MIST-net proposed a multidomain transformer model to reconstruct CT scans [82]. MIST-net can reduce radiation doses without compromising image quality. MIST-net incorporates the Swin transformer architecture, residual features, and an edge enhancement filter to reconstruct the desired CT image.

### Applications of ViT in telehealth

**Fig. 13:**
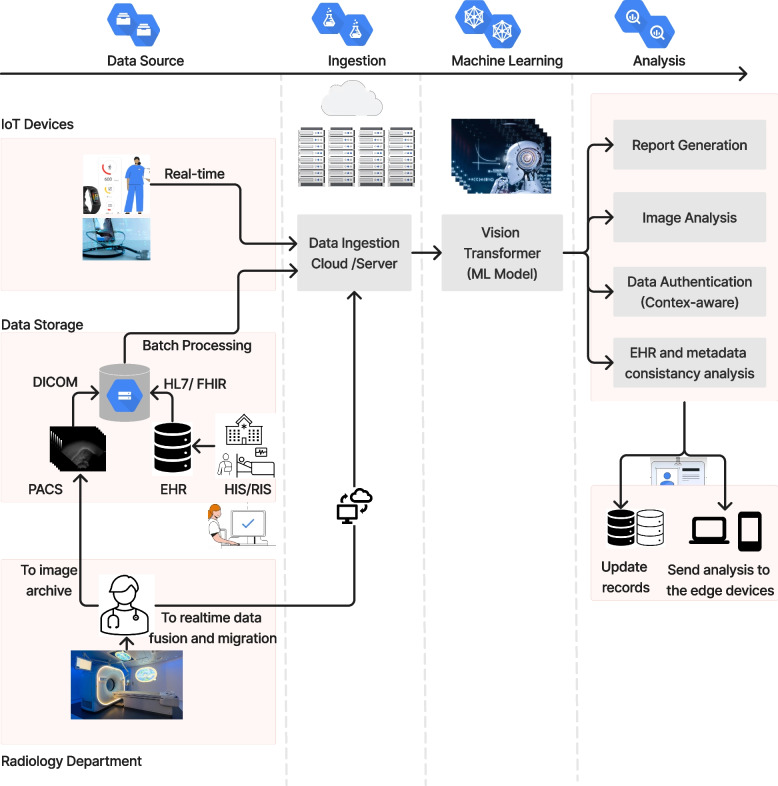
Schematic of the components of the ViT in a telehealth ecosystem

There is an increasing need for efficient techniques to process all medical information within the healthcare ecosystem. This is because of the complex nature of the unstructured format of medical data, such as images, clinical reports, and laboratory results. The ViT provides a comprehensive solution as it can process medical data in different formats and automatically generate reports or instructions. Figure 13 shows the main components of a telehealth ecosystem: the data source, ingestion, machine learning, and data analysis.

The hospital information system (HIS) and radiology information system register the patient and store data in electronic health records (EHRs) and picture archiving and communication systems (PACS) to be shared within the telehealth ecosystem. The HIS relies on standards such as Health Level 7 and Fast Healthcare Interoperability Resources for the exchange of patient metadata or EHRs [84, 85]. PACS is used to store and transfer medical images, mainly in the Digital Imaging [86] and Communications in Medicine [87] format, which are available to medical staff for further clinical analysis.

Patient data are shared in a cloud or server, either in real-time streaming or in batches from a data storage warehouse or data lake. The ViT or any other machine learning model is used to train the system on the ingested data. Once the model has been deployed, the ViT can be used to analyze medical data, approximately 90% of which are in an image format. Once the data have been analyzed, the results are sent to update patient records in the EHR or other storage systems.

#### Applications of ViT in report generation

The ViT provides a unified solution that processes text along with unstructured data, such as images. The advantage of using the ViT is that it can process and generate radiology reports, surgical instructions, and other clinical reports in a global context by retrieving huge amounts of information stored in health information systems.

**Fig. 14:**
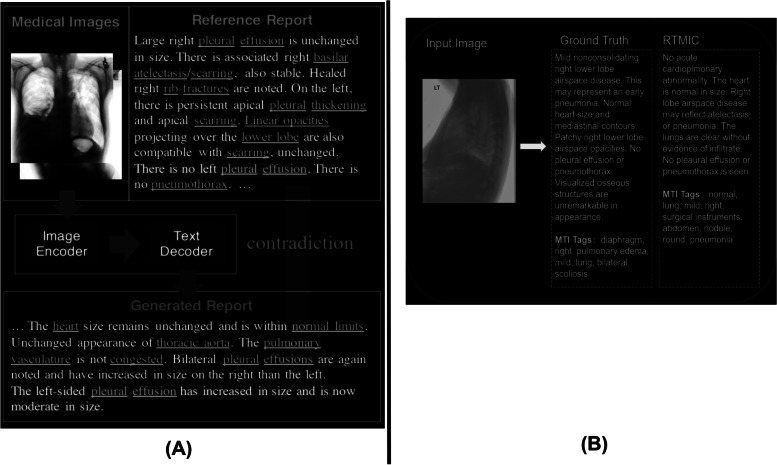
Examples of report generation from the input image using the ViT. **a** Sample of results by the IFCC algorithm [89] for report completeness and consistency; **b** Example of report generation results by the RTMIC algorithm [88]

Figure 14 shows the image capture, report consistency, completeness, and report generation by the Real Time Measurement, Instrumentation & Control (RTMIC) [88] and International Federation of Clinical Chemistry (IFCC) algorithms [89] from an input of medical images. The RTMIC is a ViT-based algorithm used for medical image captioning [88]. The GT is a manual reference written by an expert. Att2in is an attention-based method used for comparison [90]. The quality standards for health information systems state that the transferred data should be consistent and complete. The IFCC algorithm [89] improves the factual completeness and consistency in image-to-text radiology report generation. The algorithm uses a combination of transformers to extract features and RL to optimize the results.

The transformer efficiently addresses the challenges of handling biased medical data and long and inconsistent paragraphs. The AlignTransformer can produce a long descriptive and coherent paragraph based on the analysis of medical images [91]. It mainly operates in two stages. First, it aligns the medical tags with the related medical images to extract the features. Second, the extracted features are used to generate a long report based on the training data for each medical tag.

The transformer is also used to generate surgical reports during robot-assisted surgery by learning domain adaptation in the Learning Domain Adaption Surgical Robot (LDASR) [92]. The LDASR uses a transformer to learn the relationships between the desired region of interest, surgical instruments, and images to generate image captions and reports during surgery. Table 6 lists examples of ViT applications in image generation.

**Table 6:** Examples of ViT applications in medical report generation

Method	Category	Medical application
RTMIC [88]	Medical images general	Report generation from medical images (e.g., MRI, CT, PET and X-ray)
IFCC [89]	Medical images general	Medical report completeness and consistency
AlignTransformer [91]	Medical images general	Long report generation from medical images tags
LDASR [92]	Surgical robot camera	Surgical report generation

#### Applications of ViT in telehealth security

Telehealth security is receiving significant attention from healthcare providers owing to the emerging risks associated with leveraging advanced technologies such as machine learning. In healthcare, there is a serious risk of misdiagnosing a patient with the wrong disease or even diagnosing a healthy person with a disease.

**Fig. 15:**
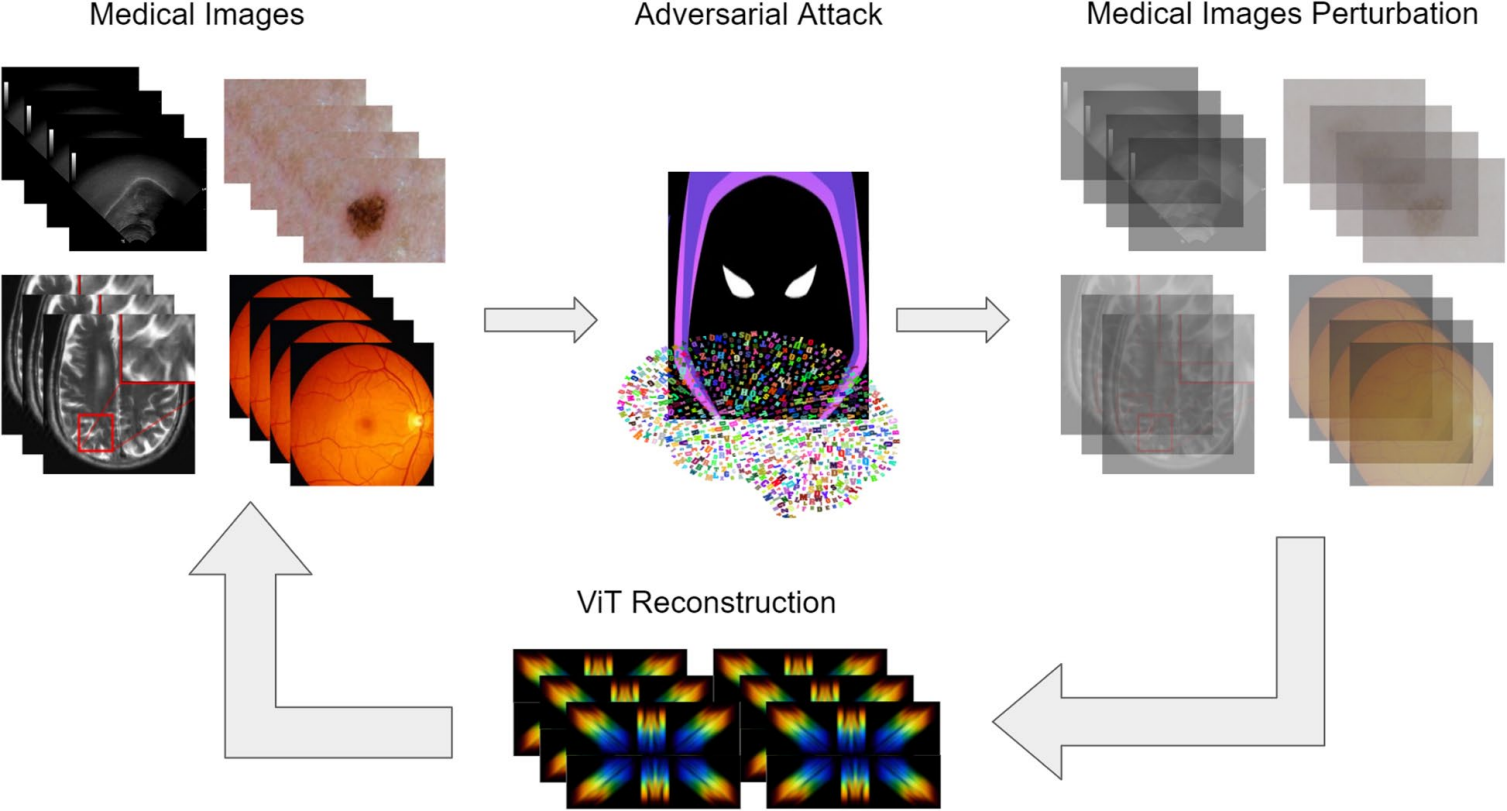
Illustration of data poisoning by an adversarial attack that fools learning-based models trained on medical image datasets

An adversarial attack refers to a malicious attack against the machine learning algorithm or data vulnerability. These attacks may include modifying the data or algorithm code, resulting in incorrect outputs [93, 94]. The accuracy of the algorithm may also be affected by the manipulation of the code or labeled data. Cybercriminals attempt to extort money from healthcare providers by threatening to publish patient information and encrypt the database. Figure 15 shows the effects of data poisoning by adversarial attacks on medical images that attempt to disrupt the behavior of the trained machine learning model.

Researchers have developed the following countermeasures against cybercrime:

1. Implement a context-aware system to ensure that the code is safe and not jeopardized.
2. Store data in an encrypted cloud environment and ensure that these are backed up.
3. Federated learning is another measure that uses a distributed computing engine to process data in geographically distributed environments that maintain data in different locations, making them difficult to hack.
4. Embrace a zero-trust policy when managing access control systems in digital health applications. This provides an additional authentication measure by considering different attributes before granting access instead of just relying on a role-based access system.

Unlike the ViT, traditional CNN-based algorithms are not robust against adversarial attacks because of the simplicity of their architecture [95]. The complexity of the ViT algorithm and its ability to extract features in a global context are solid grounds for detecting irregularities in data entry. The ViT has been used for data encryption [96], anomaly detection [97], network intrusion system detection [98], anti-spoofing [99], and patch processing [100]. Table 7 lists examples of the applications use of ViT in information system security.

**Table 7:** Examples of ViT applications in security

Method	Application
Jigsaw block-based encryption [96]	Data encryption
MFVT [97]	Anomaly detection
Image conversion from network data-flow [98]	Network intrusion system detection
Zero-shot face [99]	Anti-spoofing
Backdoor defender [100]	Patch processing

## Roadmap for implementing ViT

**Fig. 16:**
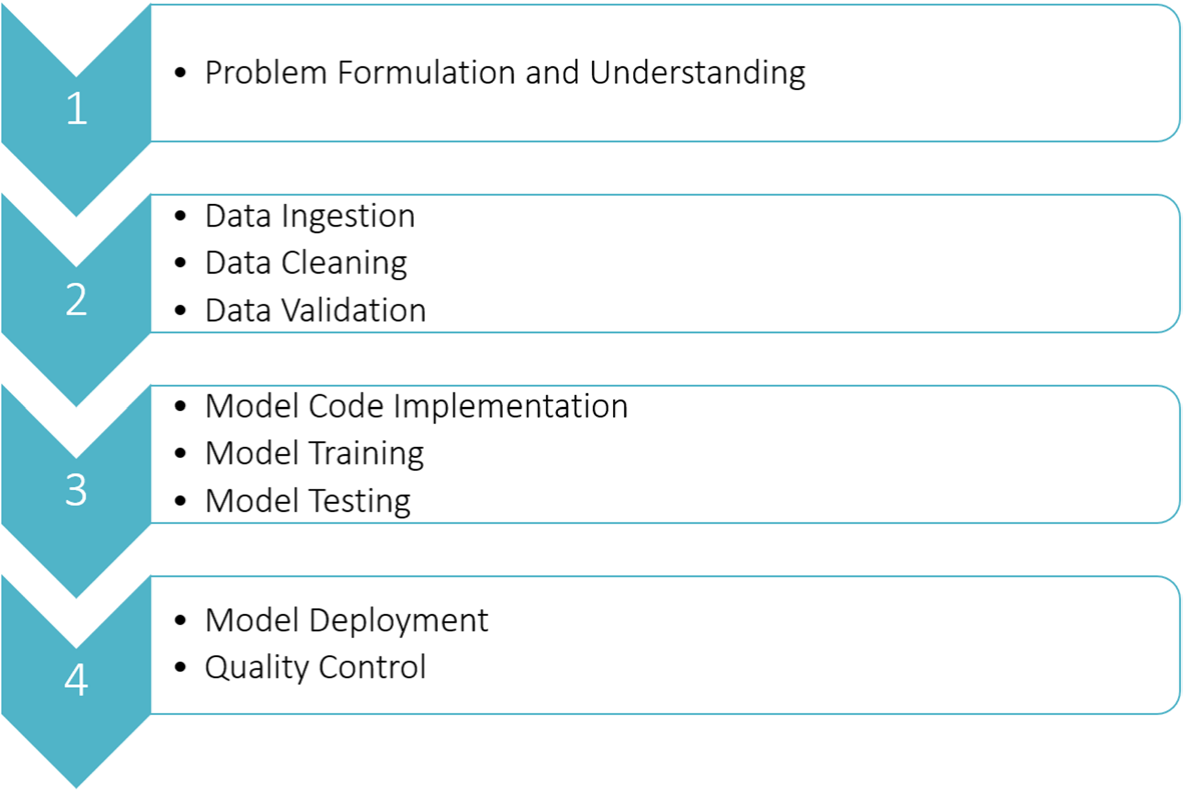
Roadmap for ViT implementation

Figure 16 shows the four stages in the end-to-end implementation of the ViT model pipeline. These are problem formulation, data processing; model implementation, training, and validation; and model deployment and quality assurance, respectively.

### Problem formulation

 Before implementing a machine learning model, the problem must be understood and formulated to fit the context of the desired product-use case.

### Data preparation

Once the problem is understood, high-quality data must be prepared for the AI algorithm. The data must be relevant, accurate, statistically balanced, and sufficient for training. The data should also be verified by different qualitative and qualitative measures to ensure their validity. This helps stabilize the model during training and speeds up convergence to obtain the optimal solution.

### Model and code implementation

There is no master algorithm that fits everything; each has its own advantages and disadvantages. The suitable ViT model or architecture is selected based on the available data and application to achieve the desired success metrics. The model hyperparameters are fine-tuned during the training stage to achieve the desired accuracy and prevent overfitting or underfitting. The model should also be validated and tested on datasets other than those used for training.

### Model deployment and testing

Finally, once the model passes all the end-to-end testing and verification processes, it should be ready for deployment. Different environments can be used to deploy the final product in different cloud or on-premise applications. The recommended environment is a cloud-based system because it can automatically generate a model on a scale that fits the computational resources for different applications. The deployed model should undergo different quality assurance and monitoring processes to ensure that the target performance of the system is met during tests outside the laboratory or development environment. Any bugs found in the code should be fixed. If the performance of the trained model is insufficient, then a new dataset should be used for training.

## Limitations and challenges of ViT in digital health

Transformer-based algorithms are emerging as the state-of-art in vision tasks to replace traditional standalone CNN architectures. However, transformer-based models have disadvantages in terms of technical or regulatory compliance requirements. These include data size and labeling, the need for a hybrid model, data bias and model fairness, and ethical and privacy challenges.

### Dataset size and labeling challenges

**Fig. 17:**
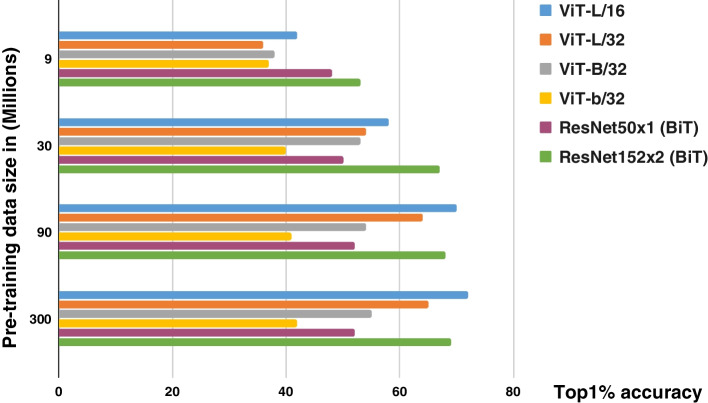
Comparison between ViT and ResNet (BiT) architecture accuracies on different sizes of training data. The *y*-axis is the size of pretraining data in the ImageNet dataset. The *x*-axis is the accuracy selected from the top 1% of the selected five-shots of ImageNet. Results according to the study in ref. [1]

**Fig. 18:**
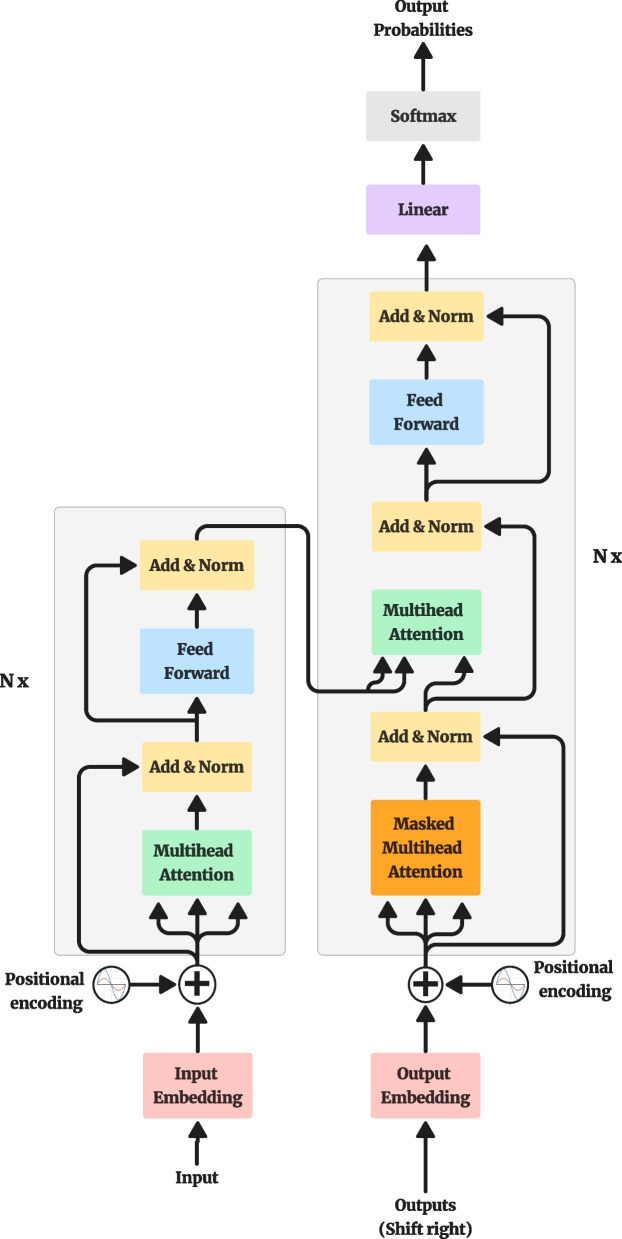
Transformer typical architecture [8]

**Fig. 19:**
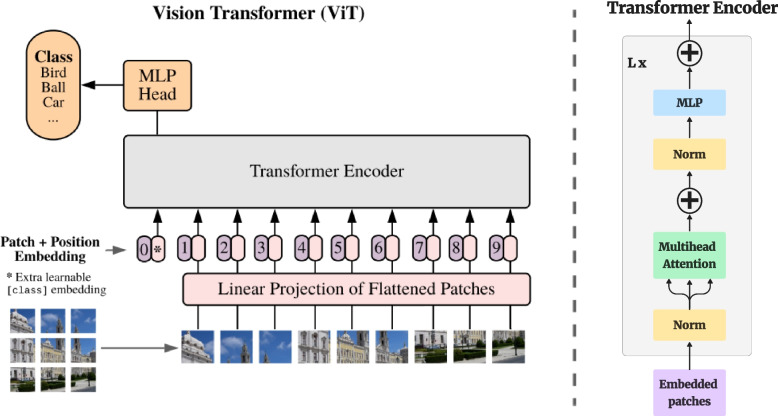
Example of using Transformer architecture for image recognition [1]

**Fig. 20:**
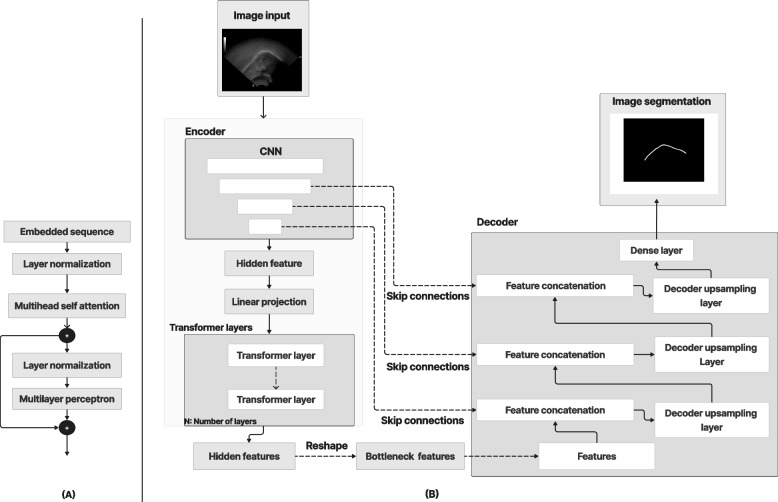
**a** Transformer layer diagram; **b** TransUnet architecture [10]

**Fig. 21:**
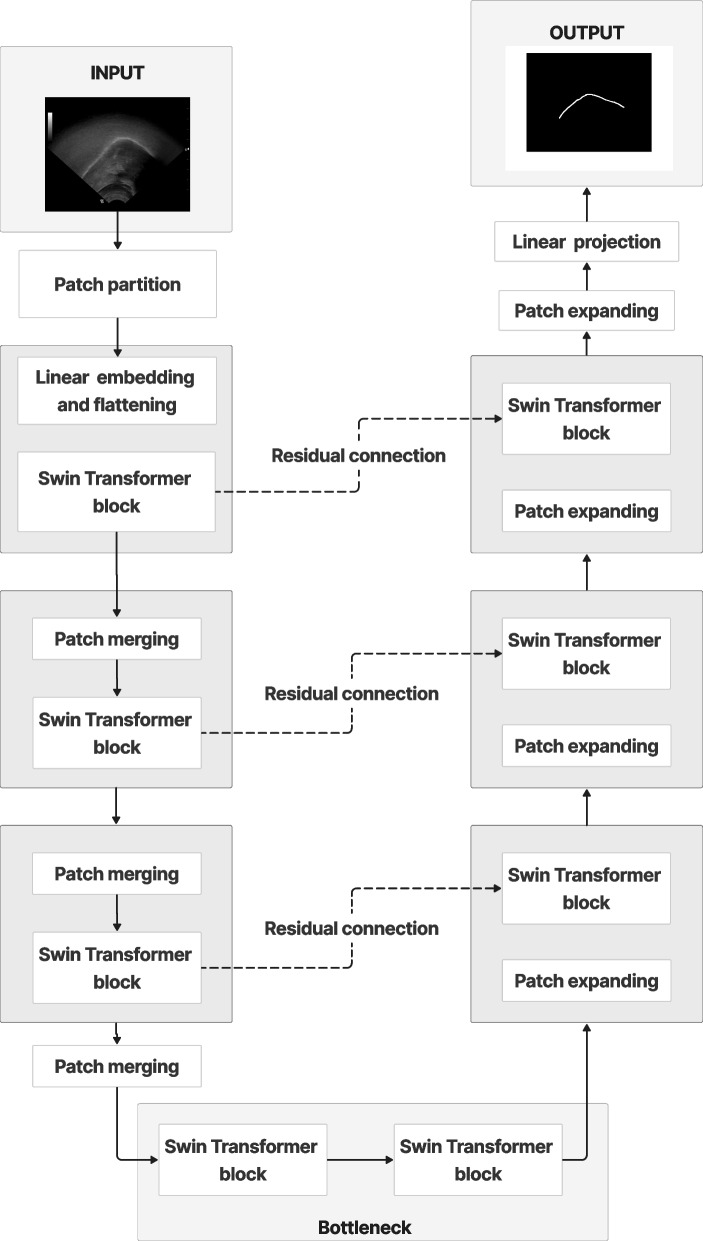
Swin TransUnet architecture [11]

Similar to other attention-based mechanisms, transformers inherently require a huge amount of data to train the model. The transformer achieved the best performance compared with the well-known ResNet architecture when trained on the JFT dataset [101], which contains 300 million images and 18000 classes. However, when trained on the ImageNet-21 k dataset [102], which contains approximately 14 million images and 21000 classes, the transformer performance did not surpass that of the ResNet architecture trained on the same dataset ImageNet-1 k [103, 104] with 1.28 million images and 1000 classes. Figure 17 shows the performance of the ViT and ResNet architectures with respect to the data size.

The results show that ResNet performed better when the dataset was small. ResNet and ViT exhibited almost the same performance when the trained on approximately 100 million samples. However, the ViT achieved superior performance compared with ResNet when the dataset size was larger than 100 million images [1].

The limited dataset size is challenging in medical applications because it is difficult to obtain a clean and high-quality dataset that is feasible for clinical application standards. Moreover, finding qualified specialists to annotate millions of images is difficult, expensive, and time-consuming.

Transfer learning, data augmentation, adversarial imaging synthesis, and automatic data labeling are among the best practices to deal with the problem of insufficient dataset size. The researchers in ref. [105] suggested that the ViT model outperformed ResNet when trained from scratch on the large ImageNet dataset without using data augmentation or a large pretrained model. Thus, there is a tradeoff between dataset size limitations and performance because having a large dataset but sufficient computational resources for training remains a challenge. The use of cloud-based data training could be a solution to limited resources. However, this is an expensive option for academia and more suitable for industrial applications. Similarly, ref. [106] proposed an effective weight initialization scheme to fine-tune the ViT using self-supervised inductive biases learned directly from small-scale datasets. This reduced the need for huge datasets for training, and hence required less computational resources.

CL is beneficial in medical image applications because it can minimize the difference between similar object representations in the latent space, while maximizing the difference between dissimilar objects [107]. CL has been used with ViT in medical histopathology to classify large images (in gigapixels) and obtain inferences to distinguish between multilabel cancer cells for classification [108].

### The need for hybrid model with transformer

The transformer was initially designed to process language models in a sequential format. Since then, it has been modified to process vision tasks by splitting the image into small patches and processing them sequentially as a text-like model. The transformer can obtain inferences about the information in a global context to capture a wide range of dependencies between objects; however, it has a limited feature localization capacity. While the standalone transformer model is sufficient for most classification tasks, in the case of image segmentation for critical medical applications that require a high-quality image, the transformer performance is insufficient and must be combined with a hybrid model.

Unet or ResNet architectures are widely used as standard models for medical image segmentation that can preserve image details owing to the nature of the encoder-decoder architecture with residual connections. However, Unet and ResNet have inherited the limitation of CNNs in failing to capture a wide range of dependencies by having only local feature extraction capabilities. TransUNet was the first architecture proposed for medical imaging segmentation that combined the transformer [10] and Unet architectures for local and global feature extraction.

The transformer was also combined with RL to generate instructions for surgical robots [62, 63]. The transformer can capture features to update the state-reward status in the RL to automate robot tasks. The RL-transformer combination has also been used in medical image captioning [88] to automatically generate medical reports within the hospital system.

### Data bias and fairness

Training machine learning models using huge datasets (in millions or billions of examples) requires resources with sufficient computational power and storage. Therefore, many algorithms tend to apply dimensionality reduction to minimize model parameters, which reduces the extracted features. This allows model training with reduced computational and memory requirements. However, there is a possibility of losing information with less representation in the feature map or dataset. Consequently, the model may be biased toward labels or classes with the largest amount of training data. The bias in the results could be significant, particularly when label balancing was not performed before training. In medical applications, rare diseases and outliers could be disregarded from the model prediction.

**Fig. 22:**
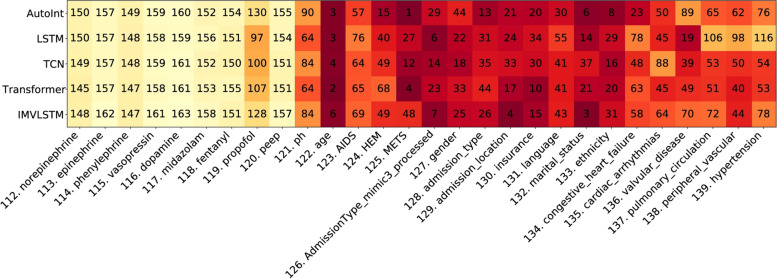
Examples of global features that are used for mortality predictions are numbered from (112-139). The numbers in the table depicts the rank sore and each column represents a feature and its importance score by different methods on the horizontal line [109]. AutoInt [111], LSTM [112], TCN [113], Transformer [8], IMVLSTM [114] are the machine learning methodologies

In ref. [109], the fairness and interpretability of DL models were evaluated using the largest publicly available dataset, the Medical Information Mart for Intensive Care, version IV. The study found that some DL models lacked fairness when relying on demographics and ethnicity to predict mortality rates. In contrast, DL models that used proper and balanced critical features for training were not biased and tended to be fair. In many models, racial attributes were used unequally across subgroups. This resulted in inconsistent recommendations on the use of mechanical ventilators for treatments or in intensive care units when relying on demographic and racial categories such as gender, marital status, age, insurance type, and ethnicity. Figure 22 in Appendix A.5 shows examples of the global features importance scores used to predict mortality rates using different machine learning methods. The figure shows the bias of the importance score toward certain features when machine learning algorithms were changed.

### Ethical and privacy challenges

Information-sharing in healthcare information systems is regulated, although privacy and ethical regulations may differ across jurisdictions. For example, the Health Insurance Portability and Accountability Act (HIPAA) of the United States regulates healthcare information systems to protect sensitive patient information. The HIPAA states that such information cannot be disclosed without patient consent. Patients also have the right to access their data, ask for modifications, and know who accesses them. While such regulations help preserve patient privacy, collecting health-related datasets or making them available to the public is a challenge. This is a critical issue in the case of the ViT as millions of examples are required to train the model and obtain accurate results. Using the ViT or any other machine learning model trained on a large dataset has a higher risk of errors, and the results are subject to ethical concerns. Many large datasets are obtained from the Internet; hence, the sources may be unknown or untrustworthy, and there is no previous consent to collect these data. Training the ViT from untrusted sources could generate false results, which could lead to errors or offensive content in generated patient reports. The consequences may be worse in the case of data breaches or cyberattacks on the healthcare information system as these could alter patient records, images, or the data streaming performance of the telehealth system. Although the ViT is more robust against adversarial attacks, there is no guarantee that the ViT-based model will not generate inappropriate content. This raises concerns regarding the need to regulate the current AI industry as well as applications in healthcare to ensure that the input and output of the systems are clean and valid for clinical applications. Federated learning from different healthcare facilities and edge devices or servers can help maintain a high level of data privacy. However, the research in ref. [110] reported vulnerabilities in retrieving original data from the shared model weights.

## Conclusions

The ViT has emerged as the state-of-the-art in image recognition tasks, replacing traditional standalone machine learning algorithms such as CNN-based models. The ViT can extract information in a global context using an attention-based mechanism and analyze images, texts, patterns, and instructions.

The superior performance of the ViT makes it practical for various digital medicine applications such as segmentation, classification, image reconstruction, image enhancement, data prognosis prediction, and telehealth security.

## Data Availability

The data underlying this manuscript is based on existing publications and is available in the referenced literature or from the corresponding authors upon reasonable request.

## Abbreviations

3D: Three-dimensional
AI: Artificial intelligence
BERT: Bidirectional encoder representations from transformers
CNN: Convolutional neural networks
COVID-19: Coronavirus disease 2019
CT: Computed tomography
CL: Contrastive learning
CRC: Colorectal cancer
COTR: Convolutional transformer
DL: Deep learning
EHR: Electronic health record
FCN: Fully convolutional network
GT: Ground truth
GAN: Generative adversarial network
HIS: Hospital information system
HIPAA: Health Insurance Portability and Accountability Act
K: Key
LSTM: Long short-term memory
MLP: Multilayer perceptron
MSA: Multihead self-attention
MRI: Magnetic resonance imaging
NLP: Natural language processing
PACS: Picture archiving and communication system
Q: Query
RL: Reinforcement learning
RNN: Recurrent neural network
V: Value
ViT: Vision transformer
DETR: Detection transformer
SATr: Slice attention transformer
UCLT: Unsupervised contrastive learning-based transformer
IHD: Intracranial hemorrhage detection
SIGT: Surgical Instruction Generation Transformer
RTMIC: Real Time Measurement, Instrumentation & Control
IFCC: International Federation of Clinical Chemistry
LDASR: Learning Domain Adaption Surgical Robot
